# Host transcriptome response to heat stress and *Eimeria maxima* infection in meat-type chickens

**DOI:** 10.1371/journal.pone.0296350

**Published:** 2024-02-23

**Authors:** Ahmed F. A. Ghareeb, James C. Foutz, Gustavo H. Schneiders, Jennifer N. Richter, Marie C. Milfort, Alberta L. Fuller, Romdhane Rekaya, Samuel E. Aggrey

**Affiliations:** 1 NutriGenomics Laboratory, Department of Poultry Science, University of Georgia, Athens, Georgia, United States of America; 2 Department of Animal and Dairy Science, University of Georgia, Athens, Georgia, United States of America; Beni Suef University Faculty of Veterinary Medicine, EGYPT

## Abstract

*Eimeria (E*.*) maxima* parasite infects chickens’ midgut disrupting the jejunal and ileal mucosa causing high morbidity and mortality. Heat stress (HS) is a seasonal stressor that impacts biological functions leading to poor performance. This study elucidates how HS, *E*. *maxima* infection, and their combination affect the ileum transcriptome. Two-hundred and forty 2-week-old males Ross708 chickens were randomly allocated into four treatment groups: thermoneutral-control (TNc), thermoneutral-infected (TNi), heat-stress control (HSc), and heat stress-infected (HSi), with 6 replicates each of 10 birds. Infected groups received 200x10^3^ sporulated *E*. *maxima* oocysts/bird, and heat-treated groups were raised at 35°C. At 6-day post-treatment, ileums of five randomly selected chickens per group were sampled, RNA was extracted and sequenced. A total of 413, 3377, 1908, and 2304 DEGs were identified when applying the comparisons: TNc vs HSc, TNc vs TNi, HSi vs HSc, and TNi vs HSi, respectively, at cutoff ≥1.2-fold change (FDR: q<0.05). HSc vs TNc showed upregulation of lipid metabolic pathways and degradation/metabolism of multiple amino acids; and downregulation of most immune-related and protein synthesis pathways. TNc vs TNi displayed upregulation of most of immune-associated pathways and eukaryotic mRNA maturation pathways; and downregulation of fatty acid metabolism and multiple amino acid metabolism pathways including tryptophan. Comparing HSi versus HSc and TNi revealed that combining the two stressors restored the expression of some cellular functions, e.g., oxidative phosphorylation and protein synthesis; and downregulate immune response pathways associated with *E*. *maxima* infection. During *E*. *maxima* infection under HS the calcium signaling pathway was downregulated, including genes responsible for increasing the cytoplasmic calcium concentration; and tryptophan metabolism was upregulated, including genes that contribute to catabolizing tryptophan through serotonin and indole pathways; which might result in reducing the cytoplasmic pool of nutrients and calcium available for the parasite to scavenge and consequently might affect the parasite’s reproductive ability.

## Introduction

Heat stress (HS) is one of the most challenging environmental stressors despite the modern climate control equipment in broiler chickens’ houses. Broiler flocks may be seasonally exposed to HS that varies in intensity according to the relative humidity of the region [1]. Multiple studies have identified the various adverse effects of acute or chronic HS on chickens, such as a severe reduction in feed intake and growth [2–4], systemic alkalosis [5], immune suppression [6, 7], and increased intestinal permeability [8]. During HS the cellular production of reactive oxygen species (ROS) is dramatically increased causing oxidative stress that impacts cellular functions and even viability [5, 9, 10].

*Eimeria* (*E*.) *maxima* is one of the most common apicomplexan species infecting chickens causing coccidiosis. *Eimeria maxima* preferably invades the upper part of the jejunum and whole ileum and produces a huge number of merogonic and gametogenic stages, damaging the intestinal mucosal lining and resulting in severe inflammation, hemorrhage, and diarrhea. Consequently, areas of erosion and ulceration would be developed exposing the intestine to secondary pathogenic invasion, such as *Clostridium perfringens* causing necrotic enteritis. *E*. *maxima* spp infection is associated with significant production losses resulting from weight loss, poor feed conversion, high morbidity, and mortality [11–13]. Exposing *E*. *maxima*-infected chickens to HS-suppressed *E*. *maxima* gametogony. Broiler chickens exposed to a combination of HS and *E*. *maxima* infection exhibited higher values for the apparent ileal digestibility and less enterocytic damage than the *E*. *maxima*-infected chickens raised under thermoneutral condition [14–16].

Identifying the differentially expressed genes (DEGs) and integrating them into known molecular pathways and biological functions could lead to the mechanisms by which cells respond to different conditions [17]. This study provides an unprecedented analysis of the ileal transcriptome under HS, *E*. *maxima* infection, and their combination to elucidate the molecular mechanisms controlling the ileal tissue response to each stressor and key molecular functions by which HS limits the *E*. *maxima* life cycle.

## Materials and methods

This study was implemented under the Animal Use Proposal (AUP) A2015 04–005 approved by the University of Georgia Animal Care and Use Committee (IACUC).

### Experimental design

Two hundred and forty 14-day-old Ross 708 male broilers were randomly allocated into four treatment groups: Thermoneutral control (TNc), thermoneutral infected (TNi), heat stress control (HSc), and heat stress infected (HSi). The thermoneutral and HS groups were raised at 20°C and 35°C, respectively. The infected groups received via oral gavage 2x10^5^ *E*. *maxima* sporulated oocysts/bird, while the control groups were mock-infected with water. *E*. *maxima* infective doses were acquired from a single oocyst cloning performed as described by Schneider *et al*. [14]. The infected groups showed obvious coccidiosis clinical signs started at 4 day-post-infection (dpi), and the beak of growth depression was at 6 dpi [16]. Only HSi group had two recorded mortalities, one was found dead at 3dpi and the other was humanly euthanized at 6 dpi. To confirm the infection, oocyst shedding was detected in the infected groups (TNi and HSi) groups, but not in control groups (TNc and HSc). All the chickens were housed in wired-floor cages and *ad libitum* supplied with water and a non-medicated standard grower diet. The human end point was considered to euthanize the bird upon showing inability to reach feed and water, sever respiratory distress, or severely emaciation. Any of these factors led to humane euthanization by cervical dislocation.

Five chickens were randomly selected from each treatment group at 6 (dpi) and euthanized by cervical dislocation to sample ~1 cm of ileum tissue from Meckel’s diverticulum. Ileum samples were snap-frozen in liquid nitrogen and then, stored at -80°C for later RNA extraction and sequencing using the NGS Illumina sequencing platform.

### Nucleic acid extraction

The trizol-chloroform method was used to extract the mRNA. Briefly, 100 mg of frozen ileum tissue was homogenized in 1 mL of trizol using Benchmark BeadBlaster Microtube Homogenizer^®^ (OVERSTOCK Lab Equipment, NH, USA), and then phase separated by mixing 0.2 mL of chloroform, 3 minutes incubation at room temperature (RT), and centrifugation (13500 xg, 4°C, for 15 min). About 550 μL of the aqueous phase was collected into a new tube containing 0.5 mL isopropanol to precipitate the RNA, mixing by vortex and 10 min incubation at RT. The mixture was centrifugated at 13500 xg, 4°C for 10 min, followed by supernatant disposal. The pelleted RNA was washed by vortex mixing with 1 mL of 75% ethanol, then reprecipitated by centrifugation at 13500 xg, 4°C for 5 min, followed by discarding the supernatants. The tubes containing the pelleted mRNA were left for 10 min at RT for drying, followed by resuspending the mRNA pellet in 100μL of nuclease-free distilled water and then incubating at 55–60˚C for 10 min before being stored at -80°C.

### cDNA library construction and sequencing

The extracted RNA was purified using the RNeasy Mini Kit (Qiagen, Hilden, Germany) following the manufacturer’s protocol. The concentration of the purified RNA was determined using a NanoDrop 2000 Spectrophotometer (Thermo Fischer Scientific, DE, USA), showing OD_260/280_ ratios for all samples >1.9. The RNA integrity number (RIN) of all samples was ≥ 9. cDNA libraries were prepared with 4 μg total RNA using the TruSeq RNA Sample Preparation Kit to acquire cDNA fragments with an average size of 229 bp, or 355 bp inclusive of the adapter sequences. The prepared cDNA libraries were sequenced by 150 bp paired-end read chemistry using the Illumina HiSeq 2000 system.

### Sequence quality control, mapping, and annotation

The quality of raw reads was assessed by FastQC, and the trimming of the low-quality reads was conducted using Trimmomatic v.0.36 [18]. The reads were aligned to the reference genome of chicken (*Gallus gallus* 5.0.90, Ensembl) using STAR aligner v.2.5.2b [19]. Hit counts were accounted for using the feature Counts v.1.5.2 package [20], considering only the unique reads for downstream analysis. Before the mapping step, the quality of reads was inspected -and trimmed when required- for the adapter sequence using Flexbar version 2.4 [21].

Sequence reads mapped to the transcriptome were reported according to their genome-equivalent coordinates. The genome coverage data were acquired using the Bamtools version 2.5.1 ’stats’ command [22]. Cufflinks version 2.2.1 [23] was utilized to detect the expressed genes and transcripts by contrasting the Tophat2 read-mapping to the Ensembl gene models for the Gallus gallus 5.0.90 assembly of the chicken genome, including the known- and predicted genes. Afterward, the fragments per kilobase of exon per million mapped fragments (FPKM) expression values for each sample were collected using Cuffdiff version 2.1.1 [23] both at the Gene (G) and gene Isoform (I) levels.

### Gene differential expression, gene ontology, and Kyoto encyclopedia of genes and genomes pathway analysis

DESeq2 [24] was used to ascertain DEGs. The Wald test within the DESeq2 package was used to acquire the p-value and log_2_ fold-change of the DEGs. The p-value then was adjusted for multiple testing with the Benjamini-Hochberg method [25]. The DEGs with false discovery rate adjusted (adj. p-value) ≤0.05 and absolute log_2_ fold change >1.2 were considered as significantly differentially expressed. The gene ontogeny (GO) analysis was conducted and functionally visualized using the Cytoscape v3.7.2 software platform provided with the ClueGO Plugin v2.5.4 application [26, 27]. The pathways analysis was performed using the Kyoto Encyclopedia of Genes and Genomes (KEGG) terms [28, 29]. The GO pathways/networks were used to classify DEGs into cellular, molecular, and biological functions.

### Hi-Seq data confirmation

The Hi-Seq expression data was confirmed using RT-qPCR. Genes were randomly selected from the common DEGs shared between different group comparisons (n = 10 genes for each comparison). The relative mRNA expression values were calculated according to 2^-ΔΔ*Ct*^ method [30], and then compared with the fold change values yielded from the Hi-Seq data (S1A–S4B Figs).

## Results

**Fig 1:**
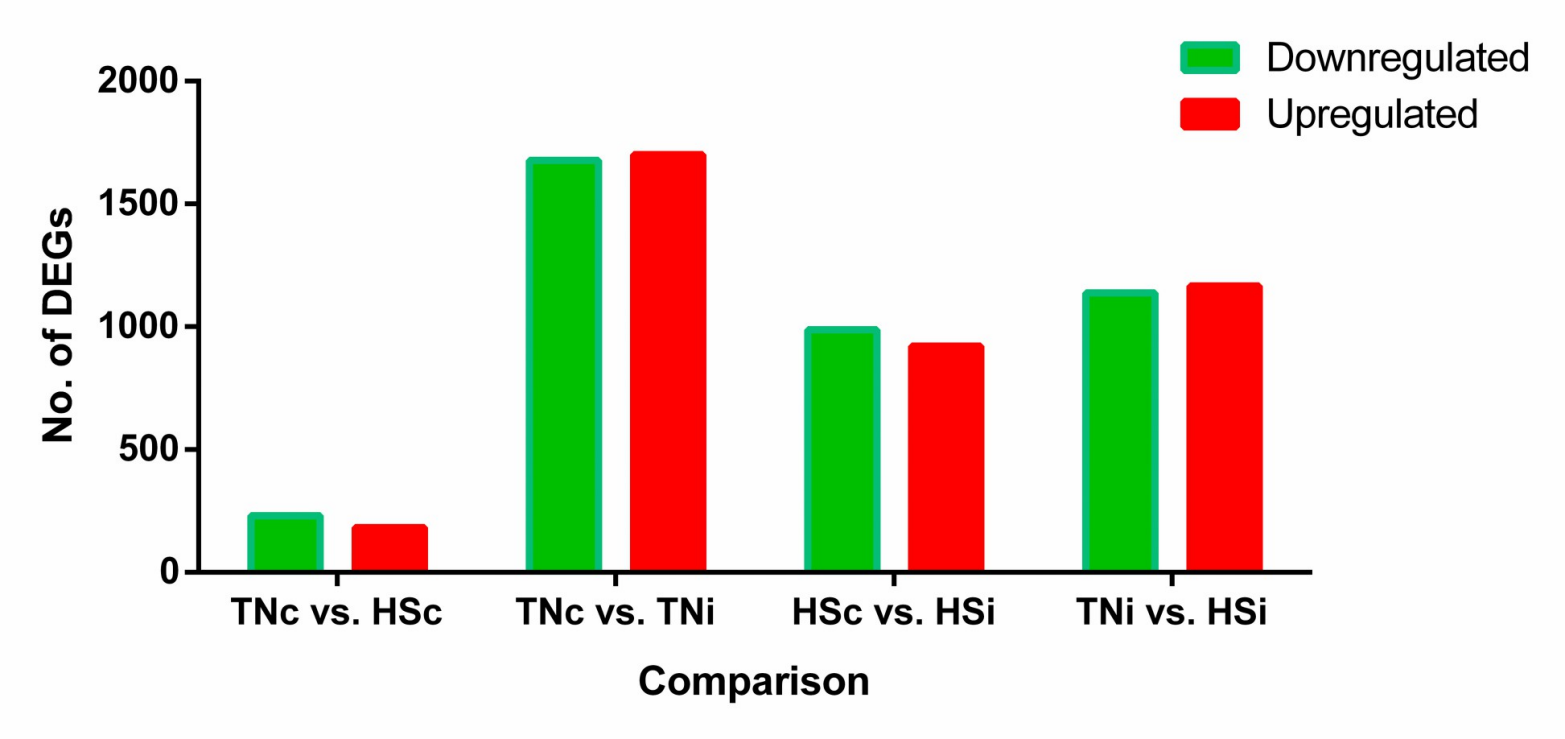
Number of DEGs of each pairwise comparison at 6 dpi of chickens infected with *Eimeria maxima* and their uninfected controls that are raised in a thermoneutral or heat stress environment: TNc = thermoneutral control, TNi = thermoneutral infected, HSc = heat stress control, HSi = heat stress infected.

The sequencing generated an average of 38,692,865 reads per sample, with an average of 32,541,154 reads uniquely mapped. The average total mapped read and unique mapped reads were 88 and 84%, respectively. The number of DEGs resulting from each pairwise comparison is presented in Fig 1. The top 100 DEGs (50 downregulated and 50 upregulated) of each pairwise comparison are provided in the supplementary Excel sheets (S1–S4 Tables).

### The effect of heat stress on the ileal transcriptome

**Fig 2:**
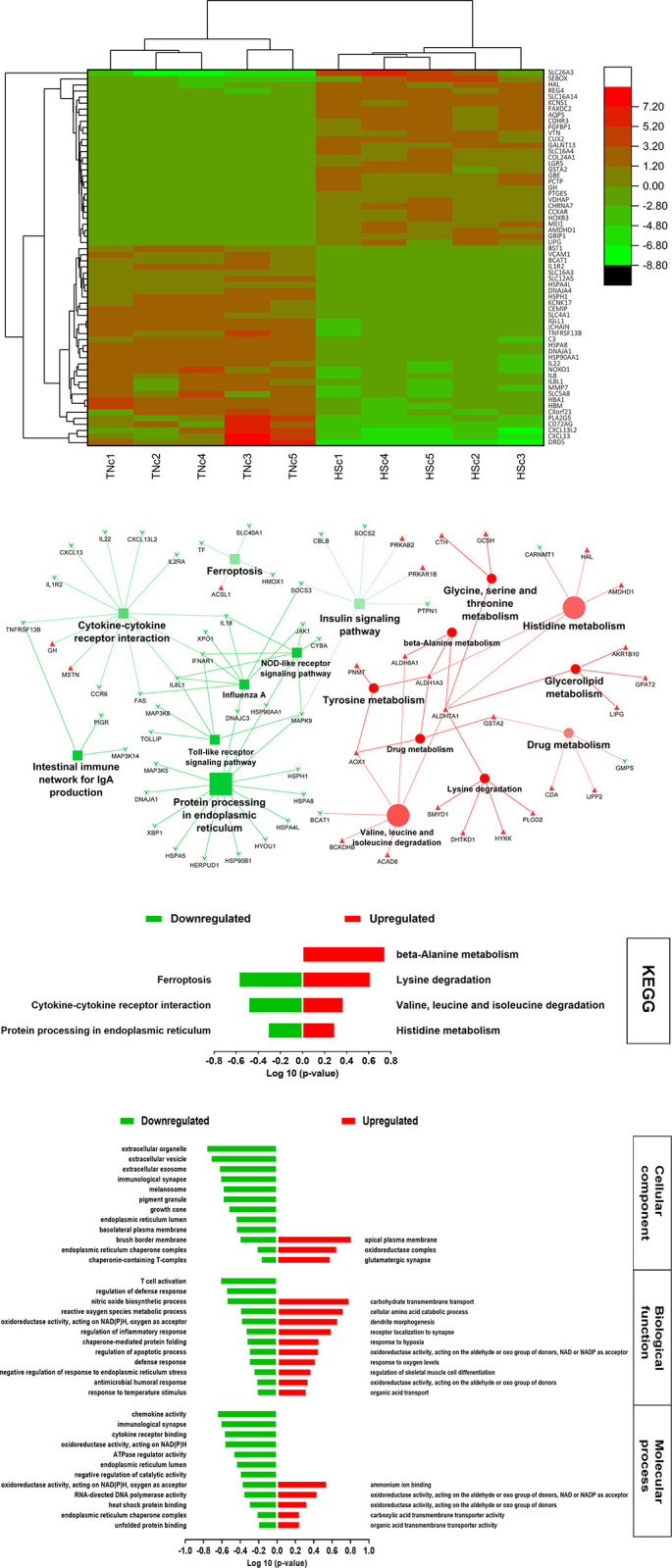
**A.** Heatmap of the top ~60 DEGs of TNc vs. HSc, calculated as Log 2 relative hit counts of the comparison between the chickens exposed to HS (HSc) and their thermoneutral control (TNc) at 6 day-post-treatment. **B.** The top KEGG pathways of TNc vs. HSc, based on the pathway term significance (α≤0.1) of the comparison between the chickens exposed to HS (HSc) and their thermoneutral control (TNc) at 6 day-post-treatment. The green squares and arrowheads depict the downregulated pathways and genes, respectively. The red circles and arrowheads depict the upregulated pathways and genes, respectively. The bigger size squares and circles denote the lower p-value. An increase in the color transparency (lighter color) of the squares and circles indicates an increase in the percentage of the included genes with expression that counter the regulation direction of the pathway. **C.** The top significant KEGG pathways of TNc vs. HSc, the comparison between the chickens exposed to HS (HSc) and their thermoneutral control (TNc) at 6 day-post-treatment. **D.** The top significant Gene Ontology terms of TNc vs. HSc, the comparison between the chickens exposed to HS (HSc) and their thermoneutral control (TNc) at 6 day-post-treatment.

A total of 413 DEGs were identified when the HSc group was compared with the TNc group (Fig 1). The genes C-X-C motif chemokine ligand 13 and ligand 13-like 2 (*CXCL13*, *CXCL13L2*); interleukins 22, 8-like 1, and 1 receptor type 2 (*IL-22*, *IL-8L1*, and *IL-1R2*); TNF receptor superfamily member 13B (*TNFRSF13B*); heat shock proteins family A (*HSP70*) member 8 and member 4 like (*HSPA8* and *HSPA4L*), family H (*Hsp110*) member 1 (*HSPH1*), and 90 alpha family class A member 1 (*HSP90AA1*) were among the most downregulated (Fig 2A) in HSc compared to the TNc group. The highest upregulated genes in the HSc group compared with the TNc group included amidohydrolase domain containing 1 (*AMDHD1*), histidine ammonia-lyase (*HAL*), cholecystokinin A receptor (*CCKAR*), cholinergic receptor nicotinic alpha 7 subunit (*CHRNA7*), polypeptide N-acetylgalactosaminyltransferase 13 (*GALNT13*), glutathione S-transferase alpha 2 (*GSTA2*), and lipase G, endothelial type (*LIPG*) (Fig 2A).

The KEGG pathway network analysis depicted in Fig 2B and 2C shows downregulation of the “cytokine-cytokine receptor interaction”, “intestinal immune network for IgA production”, “Toll- and nod-like receptor signaling”, and “protein processing in the endoplasmic reticulum (ER)” pathways, whereas the pathways involved in metabolism and degradation of many amino acids such as histidine, glycine, tyrosine, valine, and leucine were upregulated. The GO terms are exhibited in Fig 2D. The Cellular compartments showed downregulation of the terms related to “endoplasmic reticulum compartments”, “basolateral plasma membrane”, and “immunological synapse”, and upregulation of the “apical plasma membrane” (Fig 2D). Consistently, the molecular function and biological process terms related to immune response and inflammation, and protein synthesis in the endoplasmic reticulum were downregulated in the HSc group compared to the TNc group, while the terms involved in the amino acids and organic acids metabolic processing were upregulated (Fig 2D).

### The effect of *E*. *maxima* infection on the ileal transcriptome

**Fig 3:**
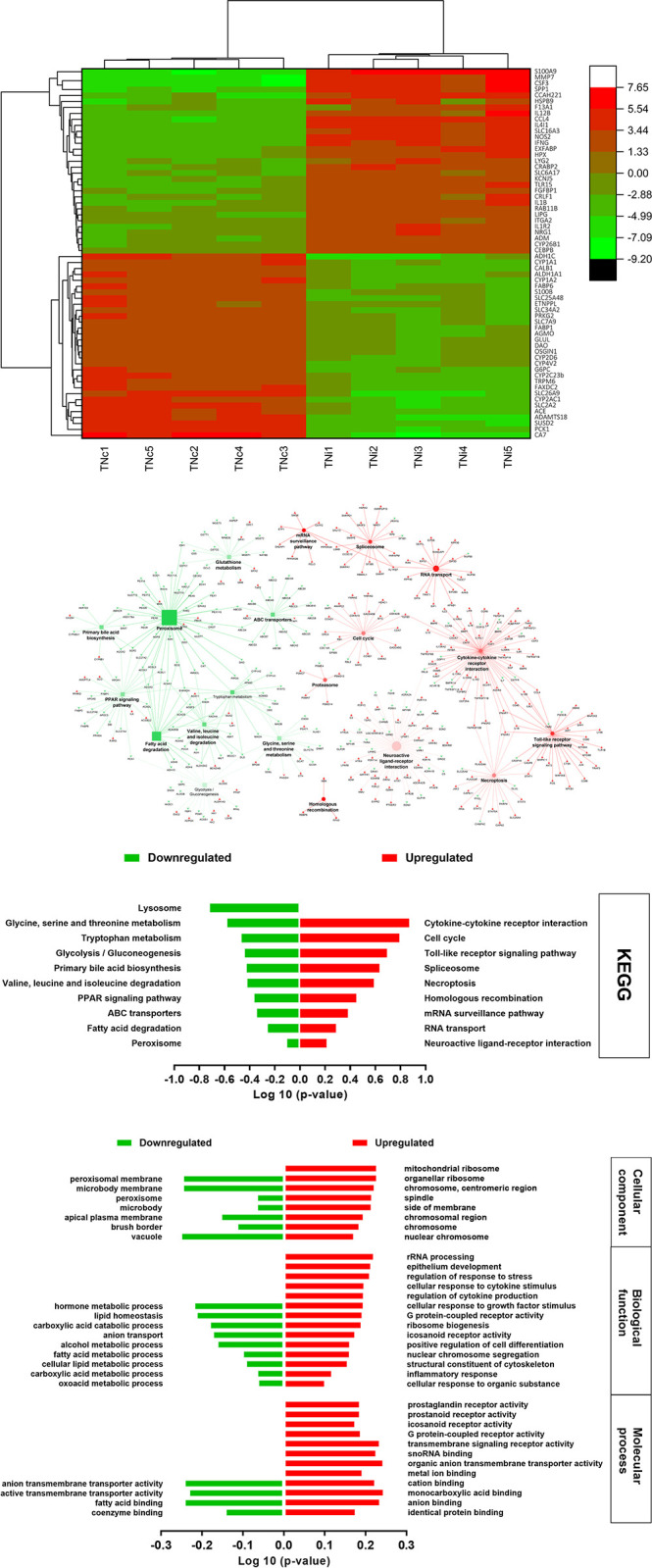
**A.** Heatmap of the top ~60 DEGs of TNc vs. TNi, as Log 2 relative hit counts of the comparison between the chickens infected with *Eimeria maxima* (TNi) and their uninfected thermoneutral control (TNc) at 6 day-post-treatment. **B.** The top KEGG pathways of TNc vs. TNi, based on the pathway term significance (α≤0.1) of the comparison between the chickens infected with *Eimeria maxima* (TNi) and their uninfected thermoneutral control (TNc) at 6 day-post-treatment. The green squares and arrowheads depict the downregulated pathways and genes, respectively. The red circles and arrowheads depict the upregulated pathways and genes, respectively. The bigger size squares and circles denote the lower p-value. An increase in the color transparency (lighter color) of the squares and circles indicates an increase in the percentage of the included genes with expression that counter the regulation direction of the pathway. **C.** The top significant KEGG pathways of TNc vs. TNi, the comparison between the chickens infected with *Eimeria maxima* (TNi) and their uninfected thermoneutral control (TNc) at 6 day-post-treatment. **D.** The top significant Gene Ontology terms of TNc vs. TNi, the comparison between the chickens infected with *Eimeria maxima* (TNi) and their uninfected thermoneutral control (TNc) at 6 day-post-treatment.

A total of 3,377 DEGs were identified when the TNi group was compared with the TNc group (Fig 1). The most downregulated genes in the TNi group compared to the TNc group included alcohol dehydrogenase 1C (class I); gamma polypeptide (*ADH1C*); aldehyde dehydrogenase 1 family member A1 (*ALDH1A1*), cytochrome P450 family 1 subfamily A members 1 and 2, family 2 subfamily AC polypeptide 1, family 2 subfamily C polypeptide 23, family 2 subfamily D member 6 (*CYP1A1*, *CYP1A2*, *CYP2AC1*, *CYP2C23b*, and *CYP2D6*); fatty acids binding proteins 1 and 6 (*FABP1* and *FABP6*); and phosphoenolpyruvate carboxykinase 1 (*PCK1*) (Fig 3A). Among the top upregulated genes were identified interleukins (*IL-1β* and *IL12B*); interleukin 1 receptor type 2 (*IL1R2*); C-C motif chemokine ligand 4 and 26 (*CCL4* and *CCAH221*); nitric oxide synthase 2 (*NOS2*); colony-stimulating factor 3 (*CSF3*), integrin subunit alpha 2 (*ITGA2*); and secreted phosphoprotein 1 (*SPP1*) (Fig 3A).

The KEGG pathway analysis shown in Fig 3B and 3C shows the downregulation of the metabolic pathways involved in cellular respiration, carbohydrate, lipids, and amino acids catabolism, while the “cytokine-cytokine receptor interaction”, “toll-like receptor signaling, cell cycle”, and “necroptosis pathways” pathways were enriched. The GO terms are depicted in Fig 3D. The cellular compartments and molecular function showed downregulation of the terms related to absorption and transportation, especially for fatty acids, and enrichment of the terms involved in producing, binding, and transporting the inflammatory signal molecules. The biological process terms related to fatty acid metabolism and lipid homeostasis were downregulated, while the terms of immunity and inflammation, including immune regulatory and signaling processes, were upregulated.

### The effect of *E*. *maxima* infection on the ileal transcriptome of heat-stressed chickens

**Fig 4:**
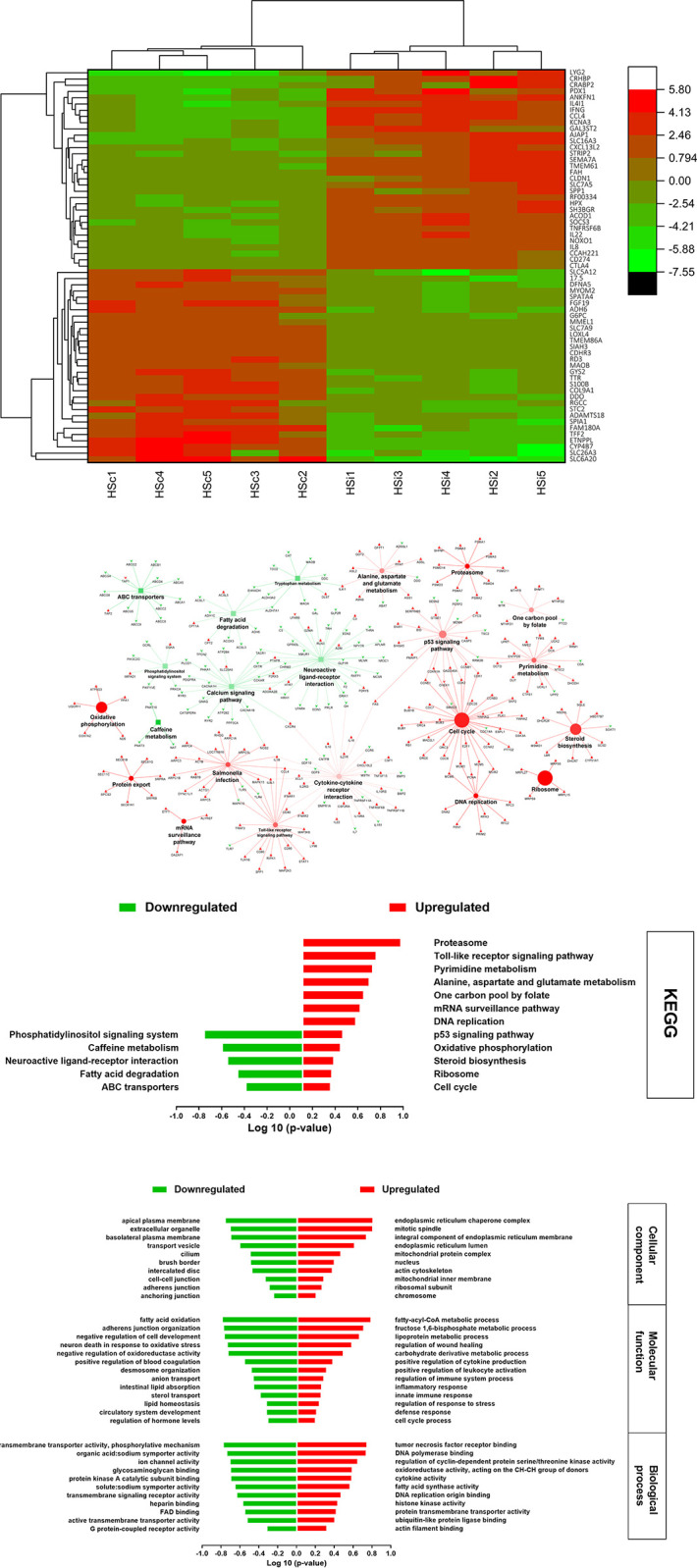
**A.** Heatmap of the top ~60 DEGs of HSc vs. HSi, as Log 2 relative hit counts of the comparison between the chickens infected with *Eimeria maxima* raised under HS (HSi) their uninfected HS control (HSc) at 6 day-post-treatment. **B.** The top KEGG pathways of HSc vs. HSi, based on the pathway term significance (α≤0.1) of the comparison between the chickens infected with *Eimeria maxima* raised under HS (HSi) and their uninfected HS control (HSc) at 6 day-post-treatment. The green squares and arrowheads depict the downregulated pathways and genes, respectively. The red circles and arrowheads depict the upregulated pathways and genes, respectively. The bigger size squares and circles denote the lower p-value. An increase in the color transparency (lighter color) of the squares and circles indicates an increase in the percentage of the included genes with expression that counter the regulation direction of the pathway. **C.** The top significant KEGG pathways of HSc vs. HSi, the comparison between the chickens infected with *Eimeria maxima* raised under HS (HSi) and their uninfected HS control (HSc) at 6 day-post-treatment. **D.** The top significant Gene Ontology terms of HSc vs. HSi, the comparison between the chickens infected with *Eimeria maxima* raised under HS (HSi) and their uninfected HS control (HSc) at 6 day-post-treatment.

A total of 1,908 DEGs were identified when the HSi group was compared with the HSc group. The top ~60 DEGs of the HSi group compared with the HSc group are exhibited in Fig 4A. The most downregulated genes in the HSi group compared with the HSc group included alcohol dehydrogenases 6 (class V) and 1C (class I), gamma polypeptide (*ADH6* and *ADH1C*), glucose-6-phosphatase catalytic subunit 1 (*G6PC*), monoamine oxidase B (*MAOB*), D-aspartate oxidase (*DDO*), glycogen synthase 2 (*GYS2*) (Fig 4A). While the genes for *IL-8*, *IL-22*, *IL-4I1*, *CCL4*, *CXCL13L2*, *TNFRSF6B*, *CTLA4*, secreted phosphoprotein 1 (*SPP1*), Programmed cell death 1 ligand 1 (*CD274*), and claudin 1 (*CLDN1*) were of the top upregulated (Fig 4A).

The KEGG analysis depicted in Fig 4B and 4C shows downregulation of the pathways “fatty acids degradation”, “tryptophan metabolism”, “ABC transporters”, and “calcium signaling”. At the same time, the immune-related pathways “toll-like receptor” and “cytokine-cytokine receptor interaction”, “cell cycle” and its related pathways, “proteasome”, and “oxidative phosphorylation pathway” were among the significantly enriched pathways in the HSi group compared with the HSc group. The GO terms are presented in Fig 4D. The molecular function and biological process of the HSi showed downregulation of “lipid absorption and homeostasis”, “calcium transmembrane transporter activity”, and “anion transport, and ion channels”. The top enriched terms were the “defense”, ‘innate immune”, and “inflammatory responses” and related terms such as “positive regulation of cytokine production” and “activity and leukocyte activation”. Also, the “DNA polymerase”, “cell cycle”, “fructose 1,6-biphosphate”, and “fatty-acyl-CoA metabolic processes” were of the top enriched terms (Fig 4D). Consistently, the GO-cellular component showed downregulation of cell junction terms, such as “cell-cell adherence” and “anchoring junctions”, and “intercalated disc”. While, the “mitochondrial compartments”, “endoplasmic reticulum”, and “nucleus” terms were enriched.

### The effect of HS on the ileal transcriptome of *E*. *maxima*-infected chickens

**Fig 5:**
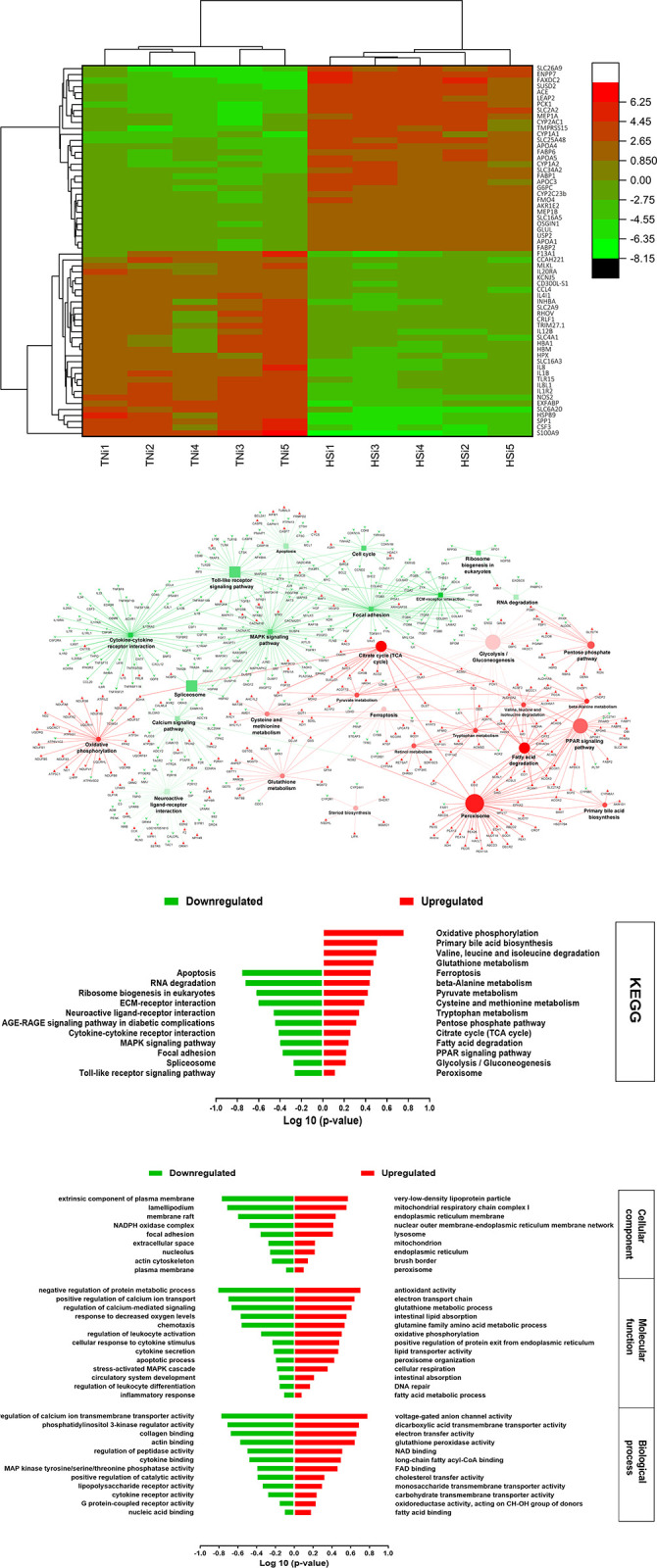
**A.** Heatmap of the top ~60 DEGs of TNi vs. HSi, as Log 2 relative hit counts of the comparison between the chickens infected with *Eimeria maxima* raised either under HS (HSi) or thermoneutral condition (TNi) at 6-day-post-treatmen. **B.** The top KEGG pathways TNi vs. HSi, based on the pathway term significance (α≤0.05) of the comparison between the chickens infected with *Eimeria maxima* raised either under HS (HSi) or thermoneutral condition (TNi) at 6 day-post-treatment. The green squares and arrowheads depict the downregulated pathways and genes, respectively. The red circles and arrowheads depict the upregulated pathways and genes, respectively. The bigger size squares and circles denote the lower p-value. An increase in the color transparency (lighter color) of the squares and circles indicates an increase in the percentage of the included genes with expression that counter the regulation direction of the pathway. **C.** The top significant KEGG pathways of TNi vs. HSi, the comparison between the chickens infected with *Eimeria maxima* raised either under HS (HSi) or thermoneutral condition (TNi) at 6 day-post-treatment. **D.** The top significant Gene Ontology terms of TNi vs. HSi, the comparison between the chickens infected with *Eimeria maxima* raised either under HS (HSi) or thermoneutral condition (TNi) at 6 day-post-treatment.

A total of 2,304 DEGs were identified when the HSi group was compared with the TNi groups. The genes interleukins 8, 1B, and 12 B (*IL8*, *IL1B*, and *IL12B*); the interleukin receptors 1R2 and 20RA (*IL1R2* and *IL20RA*), secreted phosphoprotein 1 (*SPP1*), hepatocyte growth factor, and nitric oxide synthase 2 (*NOS2*) were among the most downregulated in the HSi group compared with the TNi group (Fig 5A). The top upregulated genes included apolipoproteins A1, A5, and C3 (*APOA1*, *APOA5*, and *APOC3*); fatty acid-binding proteins 1, 2, and 6 (*FABP1*, *FABP2*, and *FABP6*); cytochrome P450 family 1 A polypeptides 1 and 2 (*CYP1A1* and *CYP1A2*); and glutamate-ammonia ligase (*GLUL*).

The KEGG pathway analysis of the HSi group compared with the TNi group is depicted in Fig 5B and 5C. The top downregulated terms were “toll-like receptor signaling”, “cytokine-cytokine receptor interaction”, “focal adhesion”, and “calcium signaling” pathways. In contrast, the highest enriched pathways were the “citrate cycle”, “glycolysis/gluconeogenesis”, “oxidative phosphorylation”, “fatty acid degradation”, “glutathione metabolism”, “tryptophan metabolism”, and “steroid biosynthesis”. The GO terms are exhibited in Fig 5D. The top downregulated molecular function and biological process terms were the “calcium ion transmembrane regulation”, “inflammatory response”, “cytokine secretion and receptors”, “collagen and actin-binding”, and “leukocytes activation and differentiation”. Whereas, “antioxidant activity”, “glutathione metabolic process”, “cellular respiration, electron transport chain”, “lipid absorption, and fatty acid metabolism” were among the top enriched terms (Fig 5D). Concordantly, the GO-cellular component analysis showed downregulation of the terms “actin cytoskeleton”, “focal adhesion”, and “membrane raft”. While the terms “mitochondrial respiratory chain complex 1”, “endoplasmic reticulum”, “lysosome, and peroxisome” were the top enriched compartments (Fig 5D).

The RT-qPCR results of the target genes selected to confirm the Hi-Seq data showed a very high similarity in the expression levels to the fold change derived from the Hi-Seq analysis (S1–S4 Figs). The correlation coefficients (r) of the RT-qPCR values versus Hi-Seq yielded fold change were 0.758, 0.857, 0.891, and 0.999 for the pair comparisons TNc vs. HSc, TNc vs. TNi, HSc vs. HSi, and TNi vs. HSi; respectively. The values reflect a very strong correlation confirming the Hi-Seq results.

## Discussion

### Ileum tissue transcriptome change in response to HS

The ileal transcriptome was reshaped to maintain resources for cellular molecular functions to cope with HS-induced oxidative stress. Ileum cells upregulated the metabolism and degradation of multiple amino acids, such as lysine, histidine, valine, leucine, and isoleucine. This may plausibly be a compensatory mechanism to maintain cellular functions and energy production under low nutrient availability created by HS [15, 16, 31–33]. The results show the upregulation of multiple aldehyde dehydrogenase (*ALDH*) genes. KEGG pathway analysis shows that *ALDH* is connecting multiple amino acid degradations and metabolism pathways (Fig 2B). The *ALDH7A1* gene encodes antiquitin enzyme (α-aminoadipic semialdehyde (α-AASA) dehydrogenase) that plays a fundamental role in lysine catabolism, the process by which lysine is broken down for energy production [34]. The enzyme α-AASA oxidizes multiple aldehyde containing molecules produced from metabolizing various amino acids by converting NAD^+^ to NADH [34]. The energy yield from each NADH is identified as 2.5 ATP produced in the electron transport chain [35]. The upregulated genes *HAL* and *AMDHD1* encode enzymes involved in histidine metabolism producing L-glutamate [36]. The *BCKDHB* gene encodes the 2-oxoisovalerate dehydrogenase E1 component beta subunit involved in valine, leucine, and isoleucine degradation by producing acetoacetate and acetyl-CoA utilized in the tricarboxylic acid cycle for energy production [37]. The upregulated gene *LIPG* possesses both phospholipase and triglyceride lipase roles that mainly hydrolyzes the phospholipids part of lipoproteins and are generally involved in glycolipid metabolism [38]. Chickens under HS conditions endure visceral ischemia along with feed intake reduction resulting in severe nutrient scarcity at the cellular level [39]. The degradation and catabolism of lipoproteins and amino acids may be a potential source for producing energy and nutrient molecules in the ileum cells of chickens reared under chronic HS.

Stress hormones (mainly glucocorticoids and catecholamine) are released under the control of the neuroendocrine system in response to HS and were found to negatively impact both innate and adaptive immune functions [7]. Furthermore, nutritional limitations induced by chronic HS results putatively in a trade-off between maintaining the cellular basic functions and the immune response [40, 41]. Heat-stressed chickens expressed downregulation in the “cytokine-cytokine receptor interaction” KEGG pathway (Fig 2B) including many chemokines, cytokines, and cytokine receptor genes such as (*CXCL13*, *CXCL13L2*, *IL22*, *IL8L1*, *IL1R2*, and *TNFRSF13B*) that play a fundamental role in the innate immune response by attracting the immune cells to the affected site and initiating the inflammatory response (Fig 2B) [42]. The “cytokine-cytokine receptor interaction” pathway includes other downregulated genes that affect the cell-mediated and adaptive immune response such as *FAS*, *IL18*, and *IFNAR1* [43, 44]. Also, the HSc group showed downregulation in gene *CCR6*, which is the key connection between the immature dendritic cells and the B cell lineage maturation for developing an adaptive immune response [45]. Consequently, the HS group show downregulation of KEGG pathways “influenza A” and “intestinal immune network for IgA production” indicating the negative regulation of both adaptive immune response compartments, cell-mediated and humeral immunity (Fig 2C & 2D).

Additionally, ER responds to cellular stress by multiple mechanisms including sensing the unfolded and misfolded proteins and then either correcting the protein folding or degrading it [46]. Chronic HS results in an increase the ROS production causing severe cellular oxidative stress that increases the rate of unfolded and misfolded protein production [47]. Eventually, ER reduces the protein synthesis to decrease the unfolded and misfolded protein production rate and also in response to reduced resources [48]. The HS group showed downregulation of the “protein processing in the ER” KEGG pathway including many downregulated genes that are responsible for recognizing the misfolded and unfolded proteins such as *DNAJC3*, *HYOU1*, and *HSP90B1* [29, 49]. The downregulated pathway also includes many genes related to ER-associated protein degradation such as *HSPH1*, *HSP90AA1*, *HSPA8*, and *HSPA4L* (Fig 2A and 2B) [29, 49]. The downregulation of the ER lumen and chaperone machinery was observed in the GO analysis terms (cellular compartments, biological function, and molecular process) (Fig 2D). Exposing chickens to chronic HS resulted in amino acid degradation for energy, and a reduction in protein synthesis and overall ER functions.

### Ileum tissue transcriptome change in response to *E*. *maxima* infection

The *E*. *maxima*-infected chickens expectedly expressed upregulation of the immune-related pathways and biological functions (Fig 3B–3D). Immune-related KEGG pathways such as “cytokine-cytokine receptor interaction”, “proteasome”, and “homologs recombination” were upregulated to establish the required innate and adaptive immune responses against *E*. *maxima* parasite (Fig 3B) [50]. The immune response is associated with increasing the transcription rate, especially for the genes encoded for inflammatory mediators, cytokines, and chemokines [51]. Therefore, there was upregulation of KEGG pathways “mRNA surveillance”, “spliceosome”, and “RNA transport” reflecting the cellular attempts for eukaryotic mRNA maturation as an initial step for protein synthesis [52]. The adaptive immune response against coccidiosis involves lymphocyte activation and homologous recombination that showed upregulation in the KEGG pathway and GO terms analysis [53].

Additionally, the “glutathione metabolism” KEGG pathway was downregulated, and some pathway-related genes were upregulated including *GPX1* and *2* which encode major enzymes that catalyze the reduction of hydrogen peroxide into water. The GPX1 and 2 enzymes contribute to protecting the cell from oxidative damage induced by *E*. *maxima* infection by catalyzing the scavenger of the oxygen radical by glutathione [54–56]. The downregulation of multiple alcohol dehydrogenase genes such as *ADH1C*, *ADH6*, *ADH4*, *ACADSB*, and *ACAA2*, acyl-CoA synthetase genes (*ACSL1* and *3*), acyl-CoA dehydrogenase (*ACADS* and *ACOX3*) that encode enzymes contributing to long and short chain fatty acid metabolism suggests the downregulation of fatty acid metabolic pathways and disturbances of cellular lipid homeostasis [57, 58]. Alteration of lipid metabolism was also reported in broiler chickens infected with *E*. *acervulina* [59] and sub-clinical necrotic enteritis [60].

### The effect of combining HS and *E*. *maxima* infection on the cellular immune functions

Comparing the HSi group with the HSc group showed a shift in the immune response related pathways triggered by *E*. *maxima* infection, since both groups were heat stressed. There was a significant upregulation in KEGG pathways “Toll-like receptor signaling”, “cytokine-cytokine receptor interaction”, and “*Salmonella* infection” suggesting that the infection might alter the previously explained immune suppression induced by HS (Fig 4B) [7]. However, the immune response seemed to be of lower expression capacity in the HSi chickens compared with the TNi chickens due to the downregulation of the “homologous combination” pathway required for lymphocyte differentiation to develop an adaptive immune response. Meanwhile, the chickens showed upregulation of the GO molecular function term for regulation of wound healing when compared with HSc (Fig 4D) but not in the TNi when compared with TNc (Fig 3D). Taken together, HSi showed an altered immune response and tissue repair functions than TNi when each was compared to its uninfected control at the same temperature level.

Comparing the HSi group against the TNi group reveals how HS altered the cellular response to the *E*. *maxima* infection and showed downregulation of all the immune response and signaling-related pathways and GO terms (Fig 5B–5D). The downregulation of the “Toll-like receptor signaling” pathway may have resulted in the reduction in the ability of the immune system to identify the *E*. *maxima* antigen and trigger an inflammatory and immune response. Consequently, all the cellular and molecular functions such as cytokine secretion and leukocytic activation contributing to the downstream cascade of immune response were downregulated. Similarly, HS diminished the immune response to *Salmonella* Typhimurium infection in broiler chickens [61]. The role of macrophages and cytotoxic (CD 8+) and peripheral lymphocytes in *Eimeria* spp sporozoite transportation through intestinal tissue laminae has been documented [62–64]. Schneider *et al*. [65] reported that HS curtailed the *E*. *maxima* life cycle and reduced gametogony in broiler chickens. Thus, it can be hypothesized that suppressing the lymphocytes and monocyte migration induced by HS might contribute to reducing the sporozoite transportation which disrupted the parasite’s life cycle leading to reducing its gametogony capacity.

### The effect of combining HS and *E*. *maxima* infection on the cellular signaling and metabolic functions

The upregulation of the “glutamate metabolism”, “pyridine metabolism”, “steroid biosynthesis”, and cell respiration-related KEGG pathways in the HSi group compared with either HSc or TNi groups may suggest improving the enterocytes functions and proliferation when the two stressors (HS and *E*. *maxima* infection) are combined rather than individually applied [66, 67]. The metabolism of both the sulfur-containing amino acids (cysteine and methionine) and glutathione was upregulated in the HSi compared to TNi (Fig 5B). The genes *GSS* and *GPX1* encoding enzymes glutathione synthetase and glutathione peroxidase1, respectively, were upregulated. Glutathione synthetase catalyzes the glutathione production form γ-glutamyl cysteine and glycine in an ATP-dependent reaction [68]. Glutathione peroxidase 1 catalyzes the glutathione antioxidant activity by scavenger ROS from hydrogen peroxide [69]. This indicates that the cellular antioxidation machinery was possibly enhanced in the HSi group compared with the TNi group. Interestingly, the molecular function GO terms showed responses to oxidative stress and low oxygen level (Fig 5D) regularly associated with HS were downregulated in the HSi group compared with the HSc group suggesting that the local hyperemia induced by *E*. *maxima* infection reversed the HS-induced intestinal ischemia.

Calcium signaling is required for the development of the protozoan *Plasmodium* spp which is closely classified with *Eimeria* spp in the phylum Apicomplexa [70]. Depleting the cell culture calcium suppressed the Plasmodium asexual stage which is restored by excess calcium supplementation [71, 72]. The results showed downregulation of molecular function GO terms “positive regulation of calcium ion transport” and “regulation of calcium-mediated signaling”, and KEGG term “calcium signaling pathway” in HSi compared to both HSc and TNi (Figs 4B, 5B and 5D). That includes the downregulation of genes *RYR2*, *CACNA1C*, and *ORAI3* encoded for ryanodine receptor 2, voltage-dependent L-type calcium channel subunit alpha-1C, and calcium release-activated calcium modulator 3, respectively. RYR is responsible for a calcium-induced-calcium release from the endoplasmic reticulum stimulated by increasing cytoplasmic calcium concentration [73]. CACNA1C is responsible for gating calcium ions current into the cell, which may activate RYR receptors [37, 74]. ORAI3 mediates calcium ions influx into the cell in response to the depletion of stored cellular calcium [75]. The catabolism of the essential amino acid tryptophan via the kynurenine pathway is necessary for the optimum development of *E*. *falciformis* in mice [65]. Tryptophan metabolism was also revealed as a metabolomic signature in *E*. *acervulina* infection [59]. The current study showed that the metabolism of the essential amino acid tryptophan was upregulated in the HSi group compared with the TNi. However, the upregulated genes mainly contribute to tryptophan metabolism through pathways other than kynurenine. For instance, the genes *DDC*, *CYP1A1*, and *CYP1A2* participate in the serotonin pathway, and the genes *MAOA*, *ALDH3A2*, *AOX2*, and *IL4I1* contribute to the indole pathway [76]. Taken together, reducing the cellular pool of nutrients and ions (tryptophan and calcium) that are scavenged by *E*. *maxima* for development and replication may explain the suppression of the parasite gametogony under HS [14, 77, 78].

### The effect of combining HS and *E*. *maxima* infection on cellular proliferation and protein synthesis

When HSi is compared with HSc, cell cycle arrest was more prominent due to the upregulation of the “P53 signaling” KEGG pathway which is one of the major cell arrest inducers (Fig 4B). P53 protein is phosphorylated under stress conditions resulting in activating the transcription of the cyclins suppressors such as *CDKN1A* and *GADD45A* that arrest the cell cycle at the S, G2 and M phases [79, 80]. That negative feedback regulation seems to be accompanied with upregulation in the genes involved in cycle regulation (Fig 4B) to enhance the immune response and tissue repair in the HSi chickens compared with HSc group [16]. The upregulation of genes upstream of the cellular apoptosis such as *FAS* and *SHISA5* enhanced the programmed death of the infected cell resulting in reducing inflammation, infection elimination, and prompt tissue repair [81]. The cell cycle pathway was downregulated in the HSi group compared with the TNi group. The upregulation of GO molecular function “DNA repair” accompanied with the upregulation of “histone kinase” which grants a negative charge to histone resulting in a higher level of chromatin opening conformation [82] suggesting that the DNA repair is enhanced in the HSi over the other groups with a single stressor.

However, KEGG pathways “ribosome biogenesis in eukaryotes” and “RNA degradation” were downregulated, the GO analysis showed upregulation of the cellular components related to the ER and the molecular function term “positive regulation of protein exit from the ER” in HSi when compared with TNi. Comparing HSi with HSc showed upregulation of KEGG pathways “ribosome” and “protein export”, and GO cellular components terms “endoplasmic reticulum chaperon complex, “endoplasmic reticulum lumen”, “endoplasmic reticulum inner membrane”, and GO molecular functions “positive regulation of protein exit from ER” suggesting that the TNi group might express a higher level of cellular and molecular functions related to protein synthesis when compared either to the TNi or HSc group.

## Conclusion

The ileal transcriptome profile was modified in response to either HS or *E*. *maxima* infection. Heat stress significantly affects the ileal transcriptome by shifting the cellular metabolic pathways from oxidative phosphorylation toward pathways exploiting the amino acids while reducing other pathways of crucial functions such as immune response and protein synthesis. Under *E*. *maxima* infection, cellular and molecular pathways involved in immune functions were enhanced to counter the parasitic invasion. However, the analysis was conducted at 6 dpi, the upregulation of pathways related to the primary immune response, such as PRR, were still prominent. Surprisingly, chickens exposed to a combination of HS and *E*. *maxima* infection showed a unique response. Both HS and *E*. *maxima* interact to modulate the cellular and molecular responses to each stressor. The HSi group showed lower expression levels of the oxidative stress-related pathways compared to the HSc group. Furthermore, the HSi group showed modifications in pathways involved in nutrient metabolism and calcium signaling compared with other treatment groups. We hypothesized that limiting the nutrients available to be scavenged by the parasite and/or hindering the sporozoite transportation by macrophages and lymphocytes may be two possible mechanisms by which HS interferes with the *E*. *maxima* life cycle.

## Supporting information

S1 Fig Genes’ expression level measured by RT-qPCR compared with the fold change produced using Hi-Seq data for the chickens exposed to HS (HSc) compared to their thermoneutral control (TNc) at 6 day-post-treatment (TNc vs. HSc) (A), and the correlation test shows the correlation coefficient (r) of the expression values produced by either method (B). Taking the expression values of the TNc as the control for the relative expression of the HSc group the expression values of the TNc group to 1 (Livak’s method). Error bars depict the SEM.
(ZIP)

S2 Fig Genes’ expression level measured by RT-qPCR compared with the fold change produced using Hi-Seq data for the chickens infected with *Eimeria maxima* (TNi) and their uninfected thermoneutral control (TNc) at 6 day-post-treatment (TNc vs. TNi) (A), and the correlation test shows the correlation coefficient (r) of the expression values produced by either method (B). Taking the expression values of the TNc as the control for the relative expression of the TNi group brought the expression values of the TNc group to 1 (Livak’s method). Error bars depict the SEM.
(ZIP)

S3 Fig Genes’ expression level measured by RT-qPCR compared with the fold change produced using Hi-Seq data for the chickens infected with *Eimeria maxima* raised under HS (HSi) and their uninfected HS control (HSc) at 6 day-post-treatment at (HSc vs. HSi) (A), and the correlation test shows the correlation coefficient (r) of the expression values produced by either method (B). Taking the expression values of the HSc as the control for the relative expression of the HSi group brought the expression values of the HSc group to 1 (Livak’s method). Error bars depict the SEM.
(ZIP)

S4 Fig Genes’ expression level measured by RT-qPCR compared with the fold change produced using Hi-Seq data for the chickens infected with *Eimeria maxima* raised either under HS (HSi) or thermoneutral condition (TNi) at 6 day-post-treatment (TNi vs. HSi) (A), and the correlation test shows the correlation coefficient (r) of the expression values produced by either method (B). Taking the expression values of the TNi as the control for the relative expression of the HSi group brought the expression values of the TNi group to 1 (Livak’s method). Error bars depict the SEM.
(ZIP)

S1 Table The top 100 DEGs (50 downregulated and 50 upregulated) of TNc vs. HSc. (XLSX)

S2 Table The top 100 DEGs (50 downregulated and 50 upregulated) of TNc vs. TNi. (XLSX)

S3 Table The top 100 DEGs (50 downregulated and 50 upregulated) of HSc vs. HSi. (XLSX)

S4 Table The top 100 DEGs (50 downregulated and 50 upregulated) of TNi vs. HSi. (XLSX)
