# Supplementary figures and images for: Host transcriptome response to heat stress and Eimeria maxima infection in meat-type chickens
Source: PLoS One. 2024 Feb 23;19(2):e0296350. doi: 10.1371/journal.pone.0296350 (PMC10889870; doi:10.1371/journal.pone.0296350)

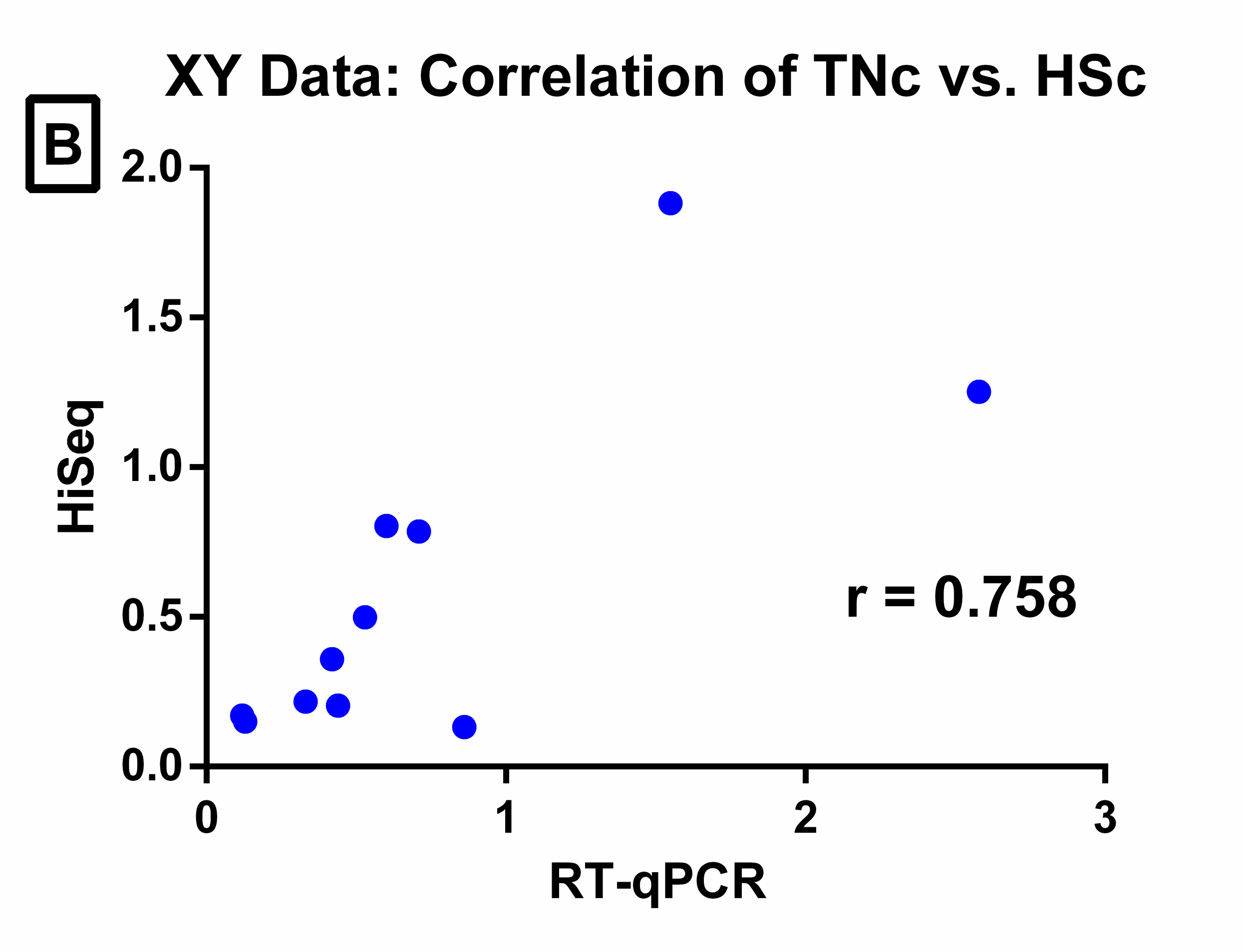

Supplement: S1 Fig — Genes’ expression level measured by RT-qPCR compared with the fold change produced using Hi-Seq data for the chickens exposed to HS (HSc) compared to their thermoneutral control (TNc) at 6 day-post-treatment (TNc vs. HSc) (A), and the correlation test shows the correlation coefficient (r) of the expression values produced by either method (B). Taking the expression values of the TNc as the control for the relative expression of the HSc group the expression values of the TNc group to 1 (Livak’s method). Error bars depict the SEM. (ZIP) [file pone.0296350.s001.zip › S1B_Fig.tif]

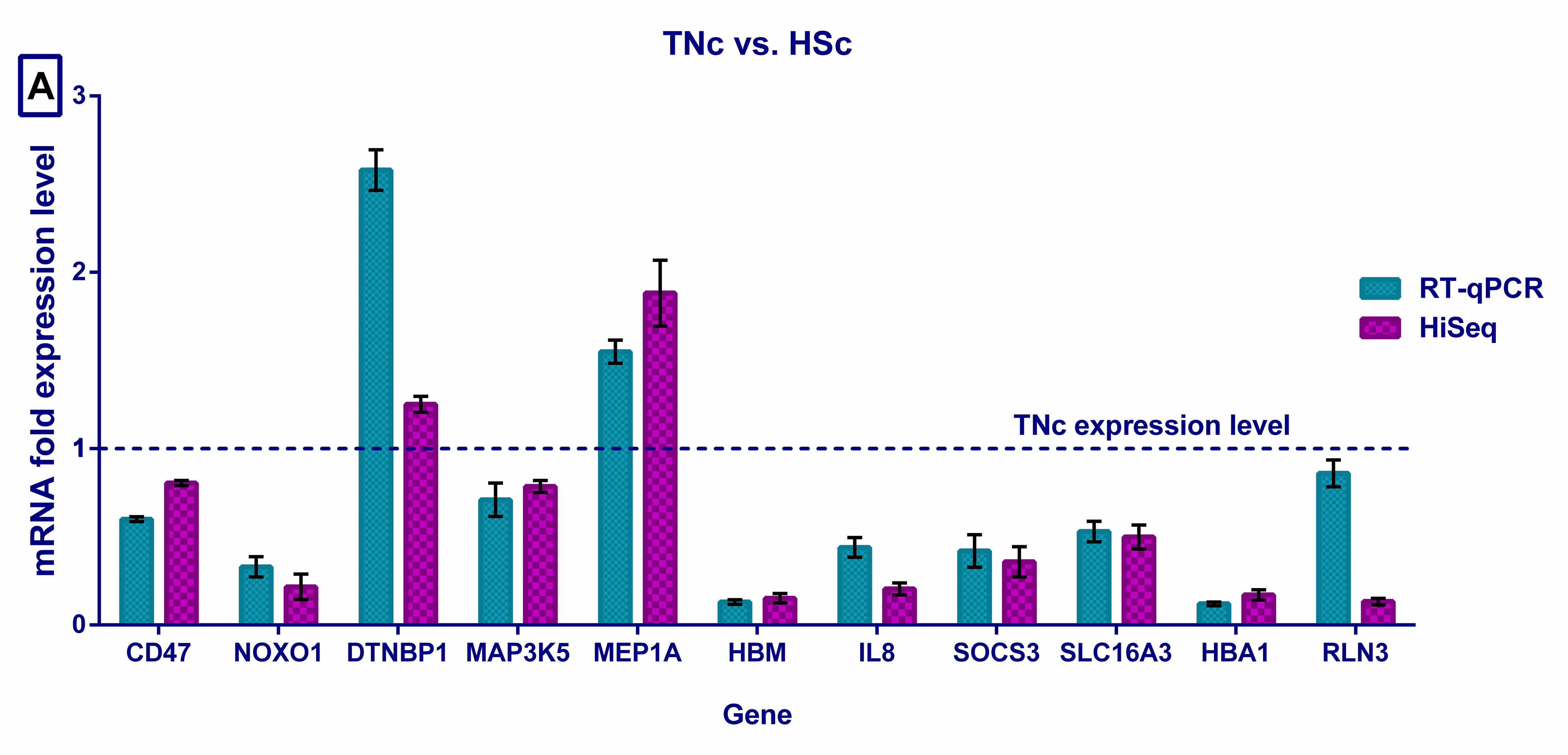

Supplement: S1 Fig — Genes’ expression level measured by RT-qPCR compared with the fold change produced using Hi-Seq data for the chickens exposed to HS (HSc) compared to their thermoneutral control (TNc) at 6 day-post-treatment (TNc vs. HSc) (A), and the correlation test shows the correlation coefficient (r) of the expression values produced by either method (B). Taking the expression values of the TNc as the control for the relative expression of the HSc group the expression values of the TNc group to 1 (Livak’s method). Error bars depict the SEM. (ZIP) [file pone.0296350.s001.zip › S1A_Fig.tif]

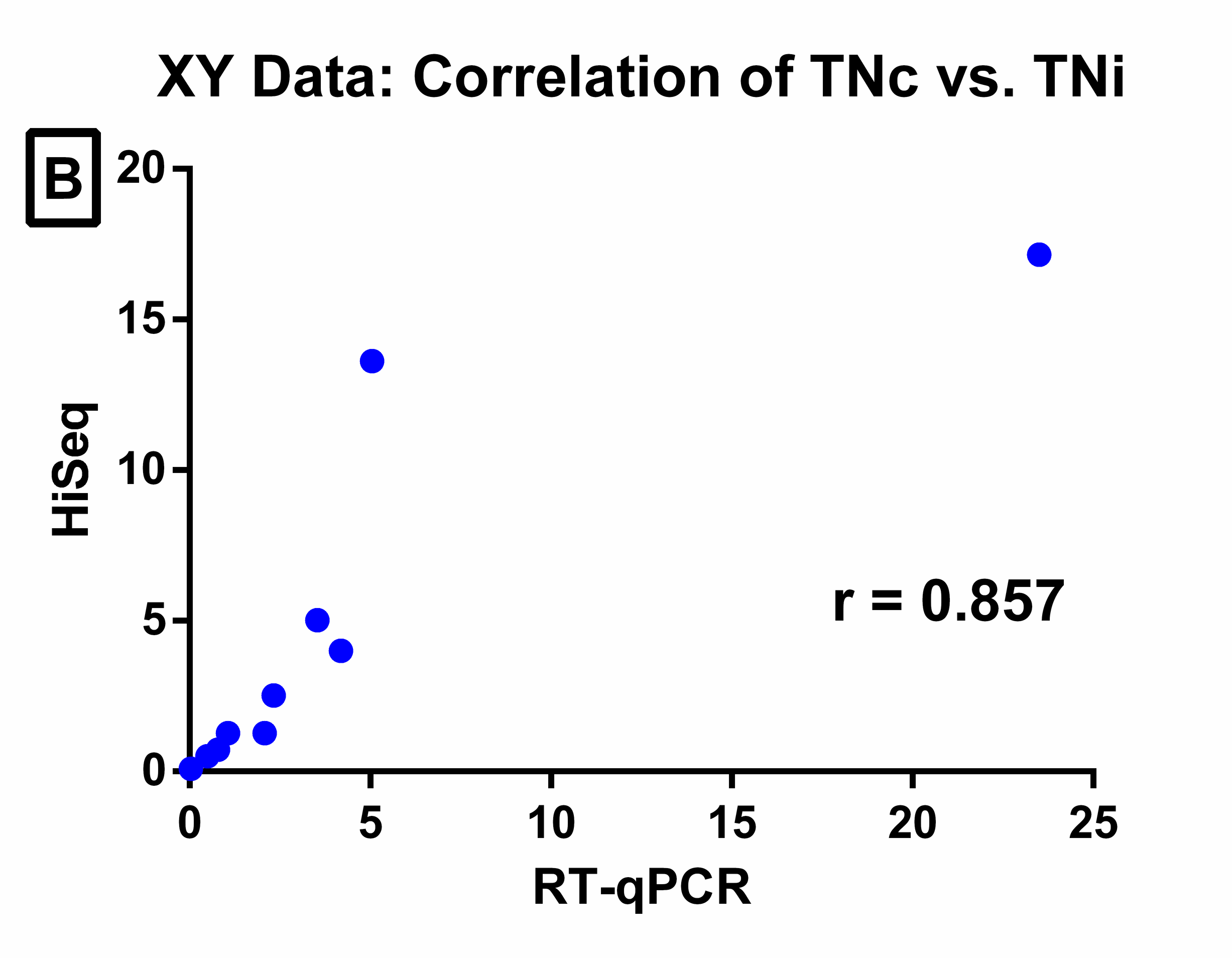

Supplement: S2 Fig — Genes’ expression level measured by RT-qPCR compared with the fold change produced using Hi-Seq data for the chickens infected with Eimeria maxima (TNi) and their uninfected thermoneutral control (TNc) at 6 day-post-treatment (TNc vs. TNi) (A), and the correlation test shows the correlation coefficient (r) of the expression values produced by either method (B). Taking the expression values of the TNc as the control for the relative expression of the TNi group brought the expression values of the TNc group to 1 (Livak’s method). Error bars depict the SEM. (ZIP) [file pone.0296350.s002.zip › S2B_Fig.tif]

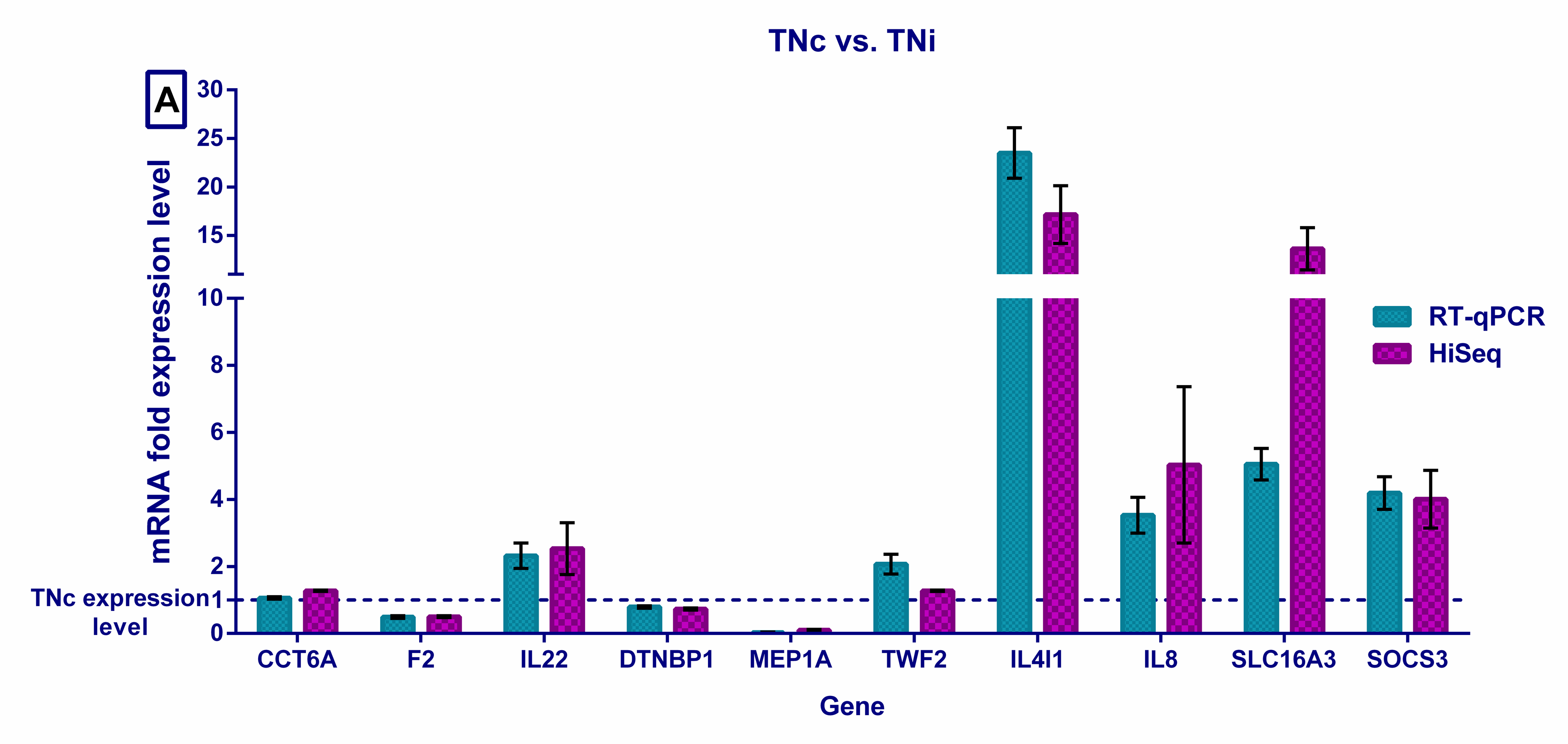

Supplement: S2 Fig — Genes’ expression level measured by RT-qPCR compared with the fold change produced using Hi-Seq data for the chickens infected with Eimeria maxima (TNi) and their uninfected thermoneutral control (TNc) at 6 day-post-treatment (TNc vs. TNi) (A), and the correlation test shows the correlation coefficient (r) of the expression values produced by either method (B). Taking the expression values of the TNc as the control for the relative expression of the TNi group brought the expression values of the TNc group to 1 (Livak’s method). Error bars depict the SEM. (ZIP) [file pone.0296350.s002.zip › S2A_Fig.tif]

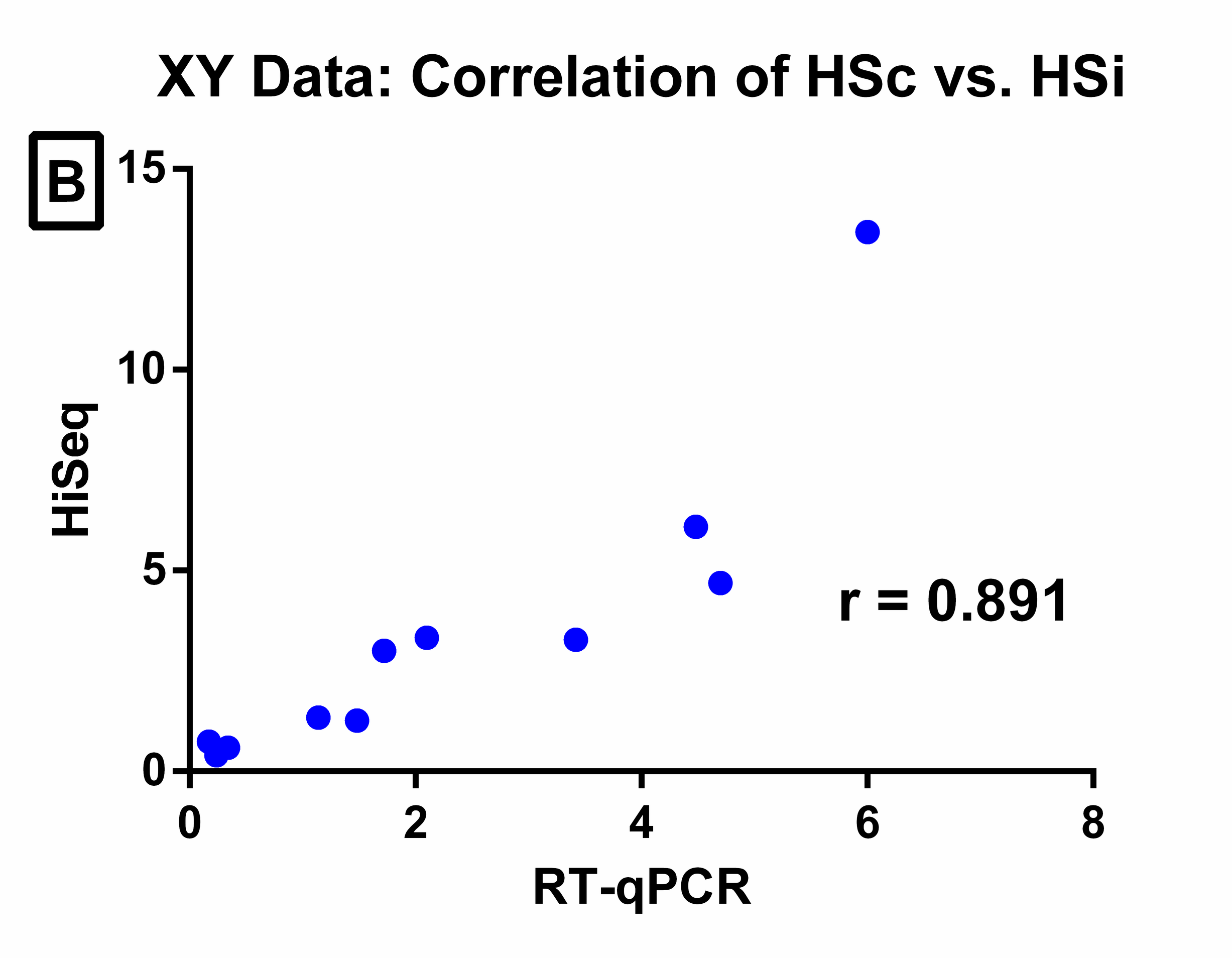

Supplement: S3 Fig — Genes’ expression level measured by RT-qPCR compared with the fold change produced using Hi-Seq data for the chickens infected with Eimeria maxima raised under HS (HSi) and their uninfected HS control (HSc) at 6 day-post-treatment at (HSc vs. HSi) (A), and the correlation test shows the correlation coefficient (r) of the expression values produced by either method (B). Taking the expression values of the HSc as the control for the relative expression of the HSi group brought the expression values of the HSc group to 1 (Livak’s method). Error bars depict the SEM. (ZIP) [file pone.0296350.s003.zip › S3B_Fig.tif]

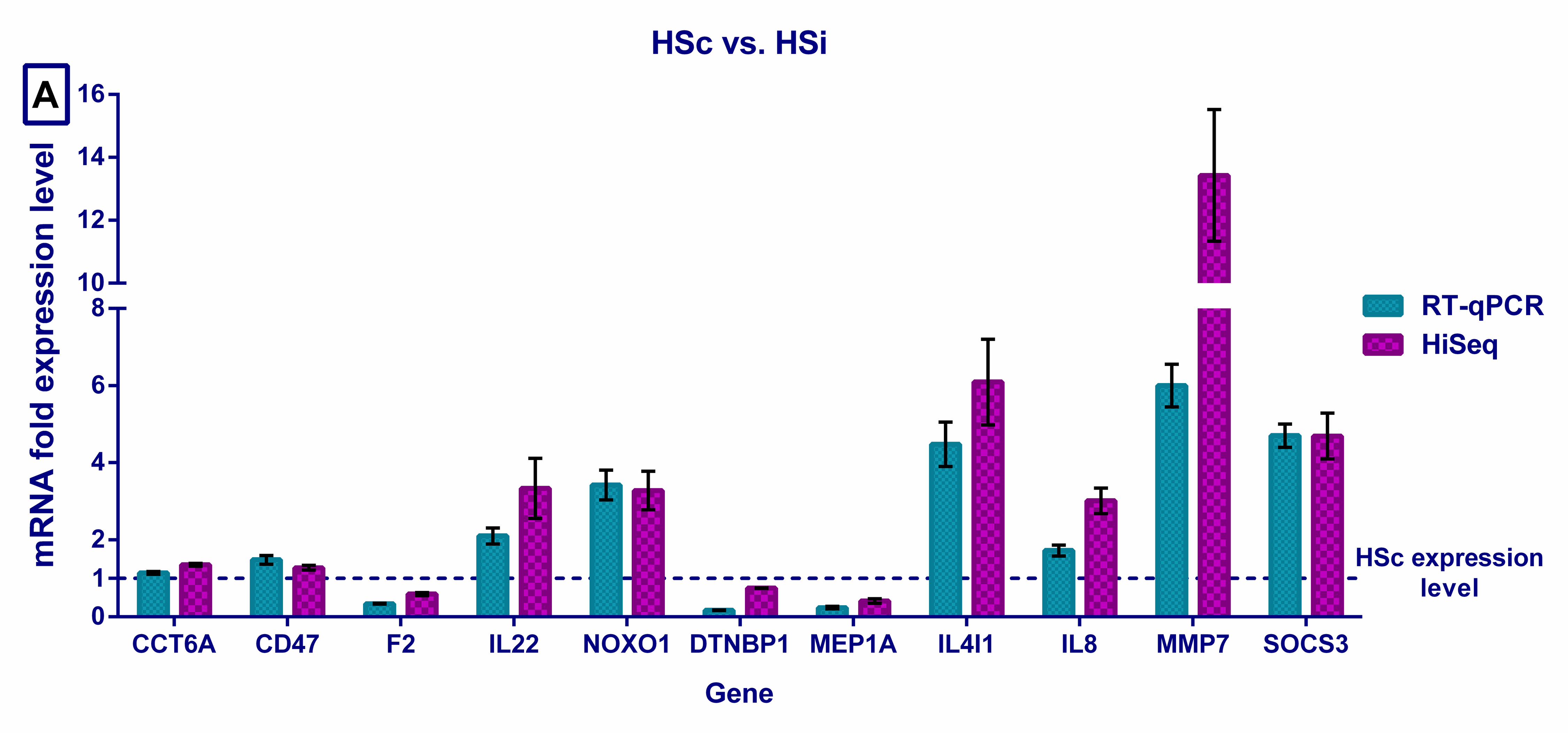

Supplement: S3 Fig — Genes’ expression level measured by RT-qPCR compared with the fold change produced using Hi-Seq data for the chickens infected with Eimeria maxima raised under HS (HSi) and their uninfected HS control (HSc) at 6 day-post-treatment at (HSc vs. HSi) (A), and the correlation test shows the correlation coefficient (r) of the expression values produced by either method (B). Taking the expression values of the HSc as the control for the relative expression of the HSi group brought the expression values of the HSc group to 1 (Livak’s method). Error bars depict the SEM. (ZIP) [file pone.0296350.s003.zip › S3A_Fig.tif]

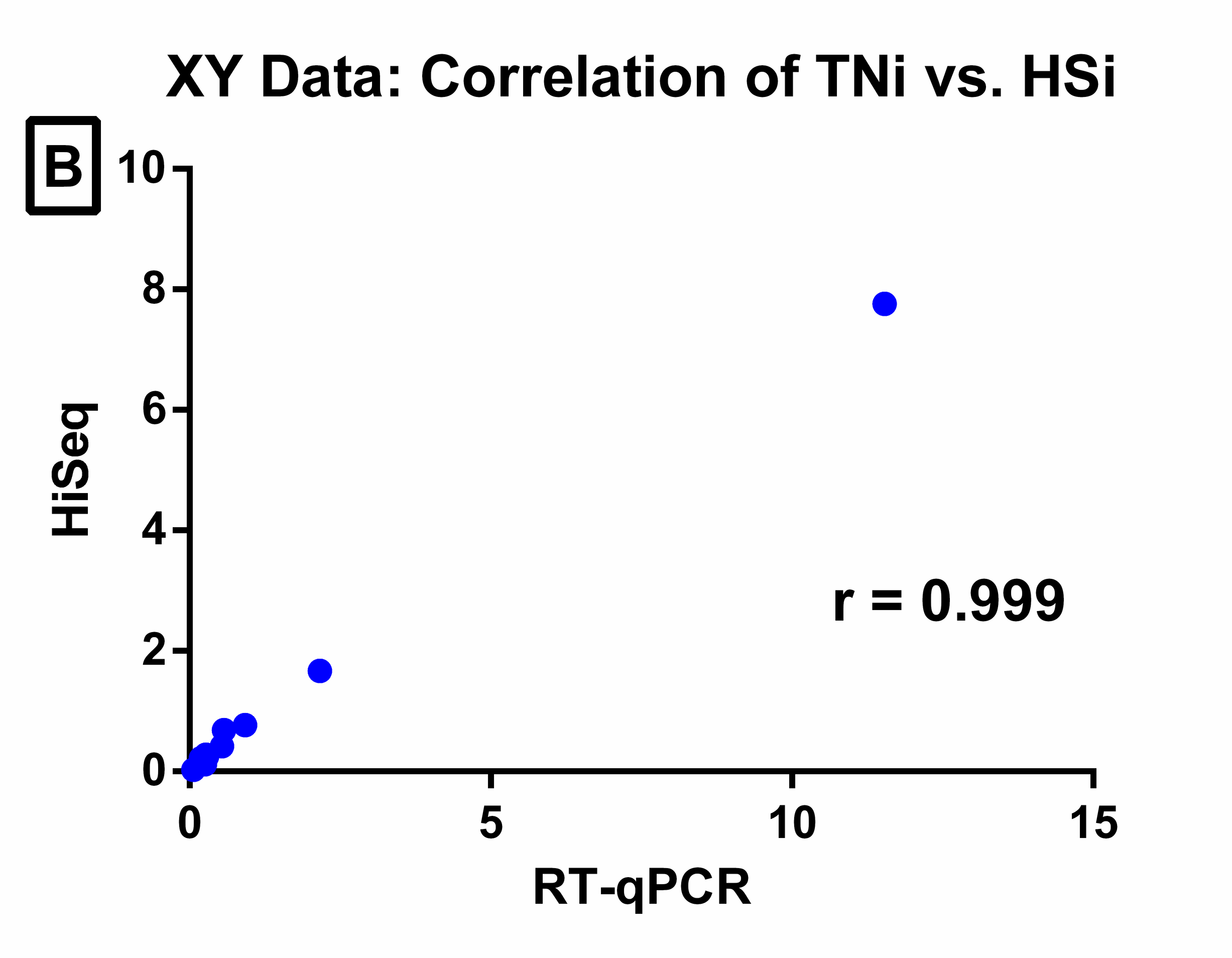

Supplement: S4 Fig — Genes’ expression level measured by RT-qPCR compared with the fold change produced using Hi-Seq data for the chickens infected with Eimeria maxima raised either under HS (HSi) or thermoneutral condition (TNi) at 6 day-post-treatment (TNi vs. HSi) (A), and the correlation test shows the correlation coefficient (r) of the expression values produced by either method (B). Taking the expression values of the TNi as the control for the relative expression of the HSi group brought the expression values of the TNi group to 1 (Livak’s method). Error bars depict the SEM. (ZIP) [file pone.0296350.s004.zip › S4B_Fig.tif]

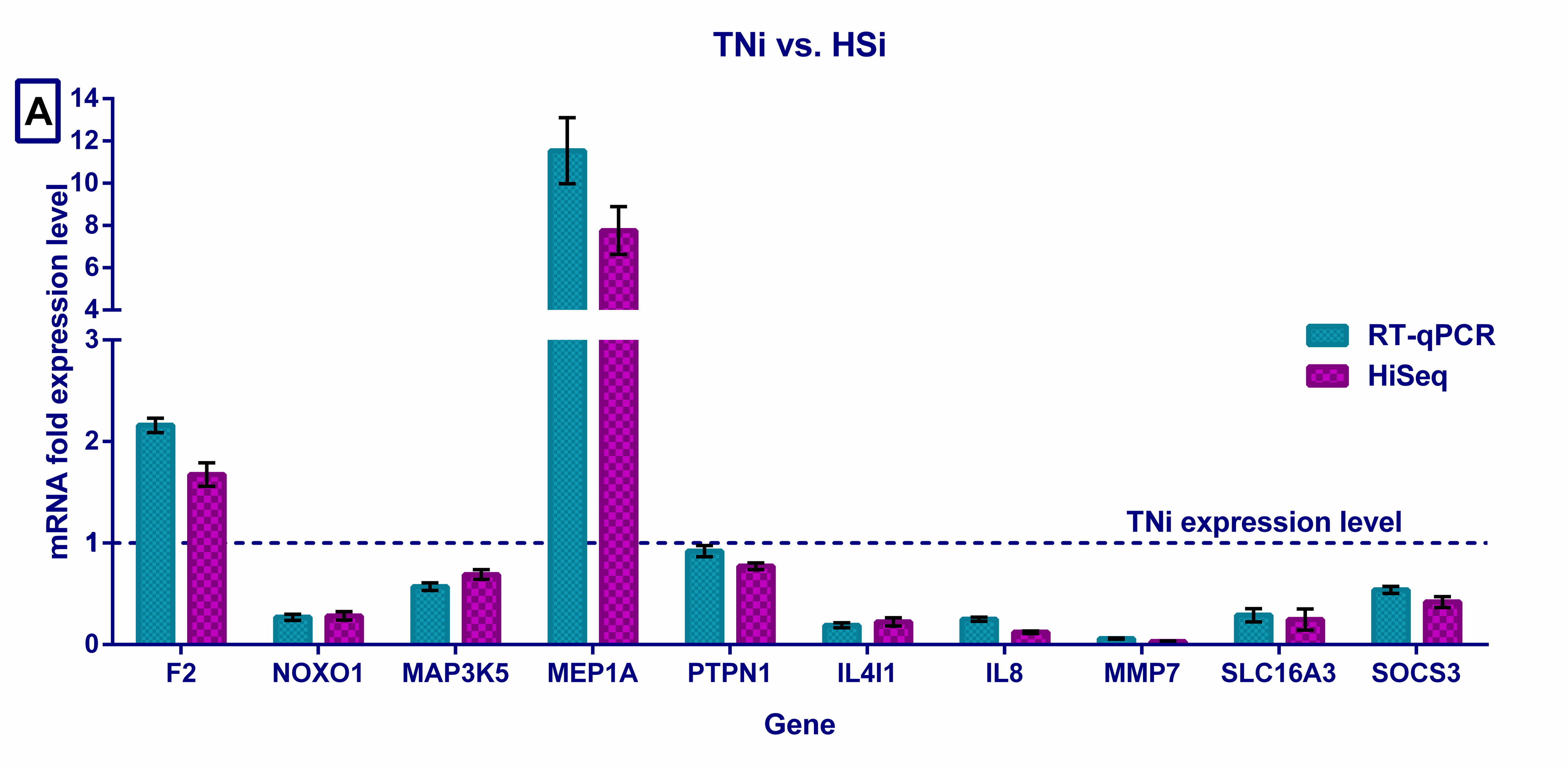

Supplement: S4 Fig — Genes’ expression level measured by RT-qPCR compared with the fold change produced using Hi-Seq data for the chickens infected with Eimeria maxima raised either under HS (HSi) or thermoneutral condition (TNi) at 6 day-post-treatment (TNi vs. HSi) (A), and the correlation test shows the correlation coefficient (r) of the expression values produced by either method (B). Taking the expression values of the TNi as the control for the relative expression of the HSi group brought the expression values of the TNi group to 1 (Livak’s method). Error bars depict the SEM. (ZIP) [file pone.0296350.s004.zip › S4A_Fig.tif]
